# Increased marrow adipogenesis does not contribute to age‐dependent appendicular bone loss in female mice

**DOI:** 10.1111/acel.13247

**Published:** 2020-10-13

**Authors:** Maria Almeida, Ha‐Neui Kim, Li Han, Daohong Zhou, Jeff Thostenson, Ryan M. Porter, Elena Ambrogini, Stavros C. Manolagas, Robert L. Jilka

**Affiliations:** ^1^ Center for Osteoporosis and Metabolic Bone Diseases University of Arkansas for Medical Sciences Little Rock AR USA; ^2^ Department of Pharmacodynamics College of Pharmacy University of Florida Gainesville FL USA; ^3^ Department of Biostatistics University of Arkansas for Medical Sciences Little Rock AR USA; ^4^ The Central Arkansas Veterans Healthcare System Little Rock AR USA

**Keywords:** aging, osteoporosis, osteoblasts, PPARγ, rosiglitazone, porosity

## Abstract

Marrow adipocytes and osteoblasts differentiate from common mesenchymal progenitors in a mutually exclusive manner, and diversion of these progenitors toward adipocytes in old age has been proposed to account for the decline in osteoblasts and the development of involutional osteoporosis. This idea has been supported by evidence that thiazolidinedione (TZD)‐induced activation of PPARγ, the transcription factor required for adipocyte differentiation, increases marrow fat and causes bone loss. We functionally tested this hypothesis using C57BL/6J mice with conditional deletion of PPARγ from early mesenchymal progenitors targeted by the Prx1‐Cre transgene. Using a longitudinal littermate‐controlled study design, we observed that PPARγ is indispensable for TZD‐induced increase in marrow adipocytes in 6‐month‐old male mice, and age‐associated increase in marrow adipocytes in 22‐month‐old female mice. In contrast, PPARγ is dispensable for the loss of cortical and trabecular bone caused by TZD or old age. Instead, PPARγ restrains age‐dependent development of cortical porosity. These findings do not support the long‐standing hypothesis that increased marrow adipocyte differentiation contributes to bone loss in old age but reveal a novel role of mesenchymal cell PPARγ in the maintenance of cortical integrity.

## Graphical abstract

Diversion of mesenchymal progenitors toward adipocytes instead of osteoblasts has been proposed to account for skeletal aging. Using mice with conditional deletion of PPARγ in mesenchymal progenitors, we show that PPARγ decreases cortical bone area but is dispensable for the loss of bone caused by PPARγ stimulators (TZD) or old age. Strikingly, deletion of PPARγ exacerbates the development of cortical porosity but attenuates osteoarthritis with aging.

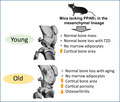

## 1 INTRODUCTION

Increased marrow fat accompanies age‐dependent bone loss, as well as the bone loss caused by estrogen deficiency and glucocorticoid excess (Meunier et al., 1971; Veldhuis‐Vlug & Rosen, 2017). Marrow adipocytes and osteoblasts arise from common mesenchymal progenitors, and commitment to each lineage occurs in a mutually exclusive fashion (Berry et al., 2015; Fan et al., 2017; Horowitz et al., 2017; Veldhuis‐Vlug & Rosen, 2017). We and others have shown that thiazolidinediones (TZDs)—activators of the critical pro‐adipogenic transcription factor PPARγ (Tontonoz & Spiegelman, 2008)—increase marrow adipocytes, decrease bone formation, and cause loss of both trabecular and endocortical bone (Ali et al., 2005; Rzonca et al., 2004; Soroceanu et al., 2004; Stechschulte et al., 2016). Further, the age‐dependent increase in bone marrow adipocytes is associated with increased lipid oxidation—a process that generates PPARγ ligands (Almeida et al., 2009), as well as increased PPARγ expression in marrow mesenchymal progenitors (Kim et al., 2017). These observations have formed the basis of the long‐standing idea that increased marrow adipogenesis concomitantly decreases the generation of osteoblasts that are needed to refill resorption cavities created by osteoclasts during the process of bone remodeling (Horowitz et al., 2017; Nehlin et al., 2019). As a result, remodeling becomes unbalanced leading to the development of involutional osteoporosis (Manolagas, 2018).

Nevertheless, marrow adipocytes may also increase bone resorption by secreting pro‐osteoclastogenic cytokines, including RANKL (Fan et al., 2017; Goto et al., 2011; Takeshita et al., 2014). Additionally, adipocytes secrete factors that promote hematopoiesis (Zhou et al., 2017) and thus may also play a role in age‐dependent changes in the hematopoietic stem cell niche (Ambrosi et al., 2017; Kim et al., 2017).

In support of the notion that PPARγ antagonizes bone formation, young mice with PPARγ haploinsufficiency exhibit increased bone mass and osteoblast number (Akune et al., 2004). In contrast, mice with conditional deletion of PPARγ in osteoblast progenitors (targeted with Dermo‐Cre, Osx1‐Cre, or 3.6kbCol1‐Cre) show either no change or a small increase in femoral or spinal bone mass (Cao et al., 2020; Sun et al., 2013). Similarly, bone mass is unaffected in mice lacking marrow adipocytes because of the deletion of 11β‐hydroxysteroid dehydrogenase (Justesen et al., 2004) or a loss of function mutation in the kit receptor (Iwaniec & Turner, 2013). It remains unknown whether PPARγ‐mediated diversion of mesenchymal progenitors to adipocytes instead of osteoblasts, or excess adipocytes, are culprits of pathologic bone loss. Herein, we investigated this issue in TZD‐treated adult mice, and in aging mice, with conditional deletion of PPARγ in mesenchymal progenitors of the appendicular skeleton. We show that mesenchymal PPARγ is dispensable for both the TZD‐induced and the age‐dependent loss of cortical and trabecular bone, but restrains the development of cortical porosity in old age.

## 2 RESULTS

### 2.1 Deletion of PPARγ from mesenchymal cells does not alter trabecular or cortical bone mass

**Figure 1:**
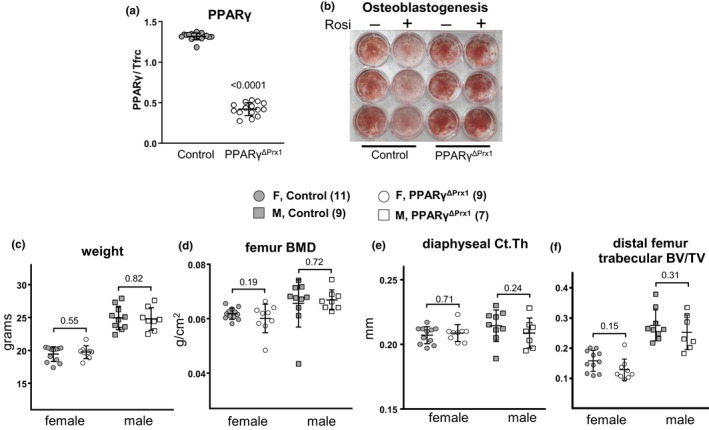
Deletion of PPARγ from mesenchymal cells has no effect on femoral cortical or trabecular bone in 3 month‐old mice. (a) PPARγ gene number levels in humeral cortical bone of control (n = 17) and PPARγ^ΔPrx1^ mice (n = 15) males. Data were analyzed by *t* test. (b) Alizarin red staining of the mineralized matrix in bone marrow stromal cells cultured from 6‐month‐old male control mice or PPARγ^ΔPrx1^ mice. (c) Total body weight, (d) BMD determined by dual‐energy X‐ray absorptiometry (DXA), and (e‐f) bone architecture by micro‐CT, of 3‐month‐old female control (n = 11) and PPARγ^ΔPrx1^ mice (n = 9), and 3‐month‐old male mice (n = 9 and 7, respectively). Data were analyzed by 2‐way ANOVA

The Prx1‐Cre transgene was used to conditionally delete PPARγ in skeletal stem cells and their progeny, including stromal support cells, multipotential progenitors, and differentiated osteoblasts and osteocytes. These are collectively designated as mesenchymal cells in this study. This transgene is active in the appendicular skeleton but not in the spine or the hematopoietic lineage (Almeida et al., 2013; Logan et al., 2002). Female PPARγ^f/f^ mice were crossed with Prx1Cre male mice to obtain PPARγ^f/f^ mice (designated control mice) and PPARγ^f/f^;Prx1‐Cre mice, designated PPARγ^∆Prx1^ mice. The level of PPARγ gene was reduced by 3‐fold in genomic DNA purified from marrow‐free humeral cortical bone of 6‐month‐old male PPARγ^∆Prx1^ mice, as compared to controls (Figure 1a). Identical results were obtained in females (not shown). PPARγ gene levels were practically identical in spleens of control and PPARγ^∆Prx1^ mice, demonstrating the specificity of the deletion and the lack of germline deletion (not shown). The inability of rosiglitazone to reduce osteoblast differentiation in cultures of marrow‐derived osteoblast progenitors from PPAR_γ_^ΔPrx1^ mice constitutes functional evidence for deletion of PPARγ (Figure 1b). In contrast, the anti‐osteoblastogenic effect of rosiglitazone was easily seen in cells from control mice, in line with previous studies (Ali et al., 2005; Almeida et al., 2009).

As expected, 3‐month‐old male mice exhibited higher body weight, bone mineral density (BMD), and trabecular bone mass of the distal femur, than females (Figure 1, Table S1; Glatt et al., 2007). Regardless of sex, deletion of PPARγ had no effect on cortical thickness (Ct.Th), trabecular bone volume (BV/TV), or the thickness, number, and separation (Tb.Th, Tb.N, Tb.Sp) of trabeculae (Figure 1, Table S1).

### 2.2 Deletion of PPARγ increases bone size in 6‐month‐old male mice, but fails to attenuate rosiglitazone‐induced bone loss

Thiazolidinediones are potent‐specific activators of PPARγ and thus represent a powerful tool to investigate whether PPARγ activation in mesenchymal osteoblast progenitors and their progeny has an adverse effect on bone homeostasis. To set the stage for this work, we first determined the impact of feeding adult male C57BL/6J (B6) mice with chow containing 150 ppm rosiglitazone for 6 weeks. As summarized in Table S2, rosiglitazone caused a greater weight gain than the control diet despite pair feeding. This was probably due to increased peripheral fat, as evidenced by a 2‐fold increase in the weight of interscapular fat. Rosiglitazone had little or no effect on trabecular BV/TV of the distal femur or proximal tibia, but it reduced Tb.Th by 8% in the femur (*p* = 0.0005) and by 5% in the tibia (*p* = 0.09). Trabecular number and Tb.Sp were unaffected. In cortical bone, rosiglitazone‐treated mice exhibited a 3%–5% decline in the thickness of the diaphyseal and distal metaphyseal femoral cortex and tibiofibular junction (*p* = 0.13, 0.03, and 0.04, respectively). Rosiglitazone also decreased trabecular and cortical bone mass in vertebral bone by 9 and 10%, respectively (*p* = 0.002 and 0.01). In a separate experiment, rosiglitazone caused a 2‐fold decrease (*p* = 0.15) in Osx1+ bone marrow cells from 4‐month‐old male Osx1‐Cre;TdRFP B6 mice, as determined by FACS (Kim et al., 2017; Figure S1). This population of cells contains bi‐potential progenitors capable of becoming adipocytes or osteoblasts (Horowitz et al., 2017; Song et al., 2012). The reduction of Osx1+cells in response to rosiglitazone in vivo is consistent with the in vitro effect shown in Figure 1b.

**Figure 2:**
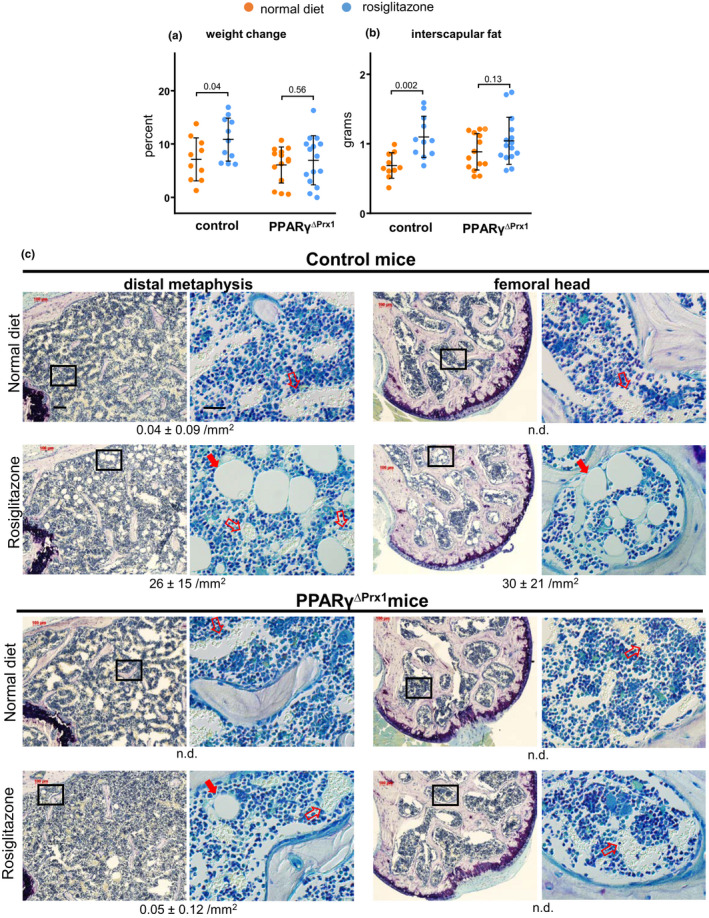
Deletion of PPARγ prevents rosiglitazone‐induced weight gain and peripheral and marrow adiposity. (a) Percent change in total body weight and (b) total interscapular fat in 6‐month‐old male mice. Controls fed normal (n = 10) or rosiglitazone (n = 11) diet; PPARγ^ΔPrx1^ mice fed normal (n = 14) or rosiglitazone (n = 15) diet. Data were analyzed by 2‐way ANOVA. (c) Photomicrographs of femoral bone longitudinal sections stained with toluidine blue. Boxes denote the region of high power image shown to the right, observed with phase illumination. Filled red arrows denote adipocytes, and open red arrows mark capillaries and vascular sinuses that contain red blood cells. Bar = 100 µm in low power images and 25 µm in high power images. Histomorphometric measurements of marrow adipocyte content are shown at the bottom of each group expressed as Ad.N per mm^2^ of marrow area, (n = 5/group), n.d., none detected

We next compared the response of 6‐month‐old male PPAR_γ_^ΔPrx1^ mice, and littermate controls, to rosiglitazone. Two‐way ANOVA results are summarized in Table S3. Administration of rosiglitazone for 8 weeks caused weight gain in control but not PPAR_γ_^ΔPrx1^ mice (Figure 2a), most likely because of deletion of PPARγ in progenitors of some peripheral fat depots as described earlier (Sanchez‐Gurmaches et al., 2015). Indeed, we observed a 60% increase in the weight of interscapular fat in control mice as compared to 18% in PPAR_γ_^ΔPrx1^ mice (Figure 2b). Adipocytes were rarely seen in marrow of the distal half of the femur, or in the femoral head, of control mice fed the normal diet (Figure 2c). As expected, there were numerous adipocytes at both of these sites in control mice fed rosiglitazone, but almost none in PPAR_γ_^ΔPrx1^ mice fed the normal or rosiglitazone containing diet.

**Figure 3:**
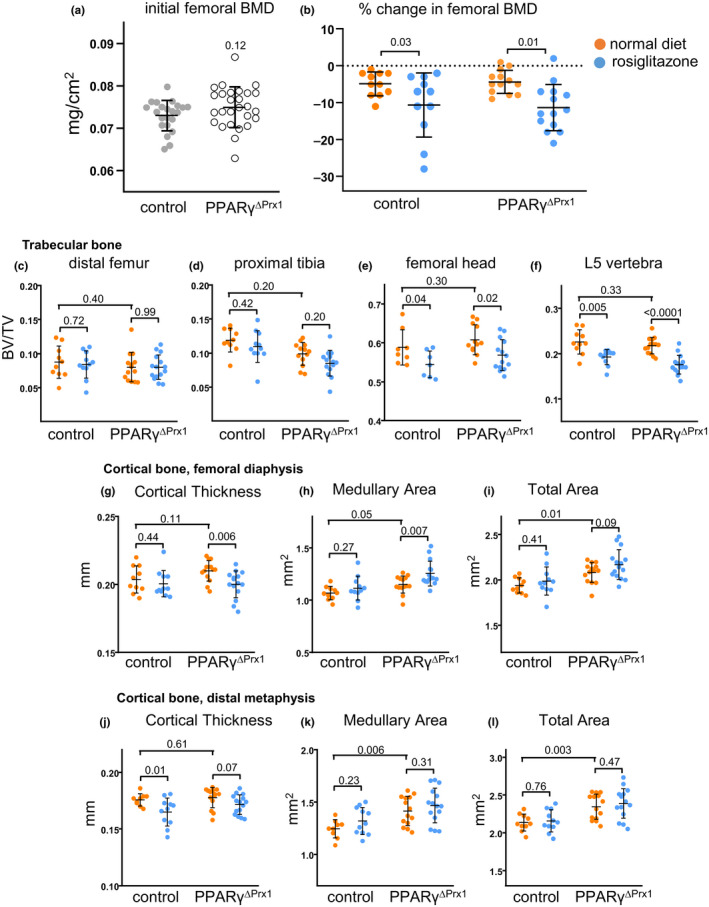
Deletion of PPARγ does not affect the magnitude of rosiglitazone‐induced loss of bone mass. (a) Baseline BMD of 4‐mo‐old male control (n = 22) and PPARγ^ΔPrx1^ (n = 27) mice. Data analyzed by Student’s *t* test. (b) Rosiglitazone‐induced change in BMD in control mice fed normal diet (n = 11) or rosiglitazone (n = 11), and in PPARγ^ΔPrx1^ mice fed normal diet (n = 13) or rosiglitazone (n = 14). Data analyzed by 2‐way RMANOVA. (c‐l) Micro‐CT measurements of indicated bones. Data analyzed by 2‐way ANOVA

PPAR_γ_^ΔPrx1^ mice fed a normal diet exhibited a 4% increase in femoral BMD as compared to littermate controls (*p* = 0.12) (Figure 3a). Micro‐CT measurements showed that PPARγ deletion had no effect on trabecular BV/TV of the distal femur, proximal tibia, or femoral head (Figure 3c,e). Thus, effect sizes were small and *p* values, for the most part, were >0.30, as detailed Table S3. The exception was Tb.Th in the femoral head which increased by 7% (*p* = 0.05) in PPAR_γ_^ΔPrx1^ mice.

PPARγ deletion had no effect on Ct.Th measured at the femoral diaphysis (Figure 3g). Nevertheless, bone size increased by 8% (*p* = 0.01), as measured by total area (Tt.Ar) (Figure 3i) and a corresponding increase in medullary area (Me.Ar) (Figure 3h). Similarly, periosteal perimeter (Ps.Pm) and endosteal perimeter (Ec.Pm) were increased by PPARγ deletion (Table S3). Changes in these cortical dimensions were also noted at the distal metaphysis (Figure 3j‐l). At the tibiofibular junction, Ct.Th was increased by 5% with PPARγ deletion (Table S4); however, the architectural basis could not be determined. Cortical porosity at the distal metaphysis of the femur was low and unaffected by PPARγ deletion, consistent with the absence of noticeable changes in volumetric BMD at this site or at the diaphysis (Table S3). Femoral length was unaffected by the deletion of PPARγ (control, 15.85 ± 0.21 mm; PPAR_γ_^ΔPrx1^, 15.84 ± 0.31 mm, *p* = 0.93).

Rosiglitazone administration caused loss of femoral BMD in both strains, amounting to 6% in controls and 7% PPAR_γ_^ΔPrx1^ mice (*p*‐int = 0.77) (Figure 3b). At the architectural level, rosiglitazone caused an 8% decrease in trabecular bone (BV/TV) in the femoral head of control mice and a 7% decline in PPAR_γ_^ΔPrx1^ mice (*p* = 0.04 and 0.02, respectively) (Figure 3e). The decrease was associated with reduced Tb.Th, whereas Tb.N and Tb.Sp were unaffected (Table S3). Equivalent structural changes were observed in control and PPAR_γ_^ΔPrx1^ mice as indicated by *p*‐int values >0.30. On the other hand, rosiglitazone had no effect on trabecular bone of the femoral distal metaphysis or the tibial proximal metaphysis of either strain (Figure 3c,d; Table S3).

Rosiglitazone had little effect on the Ct.Th of the femoral diaphysis of control mice but caused a 5% loss in PPAR_γ_^ΔPrx1^ mice (*p* = 0.006), associated with a 9% increase in Me.Ar (*p* = 0.007) (Figure 3g,h). At the distal metaphysis, Ct.Th declined by 6% in control mice and by 3% in PPARγ^ΔPrx1^ mice (*p* = 0.01 and 0.07). There was no evidence for mesenchymal PPARγ dependence of rosiglitazone‐induced changes at either of these cortical sites (Table S4). Rosiglitazone had no effect on cortical porosity in either strain (Table S3). As expected, the magnitude of rosiglitazone‐induced trabecular bone loss in the lumbar vertebra, in which mesenchymal PPARγ was not deleted, was similar in control and PPAR_γ_^ΔPrx1^ mice (Figure 3f). Overall, these findings show that deletion of PPAR_γ_ in mesenchymal cells abolished TZD‐induced adipogenesis but, if anything, intensified rather than reduced the negative impact of rosiglitazone on the appendicular skeleton of male mice. Unexpectedly, however, PPARγ deletion increased femoral bone size.

### 2.3 Aged PPARγ^ΔPrx1^ mice lack marrow adipocytes and have decreased subcutaneous fat

**Figure 4:**
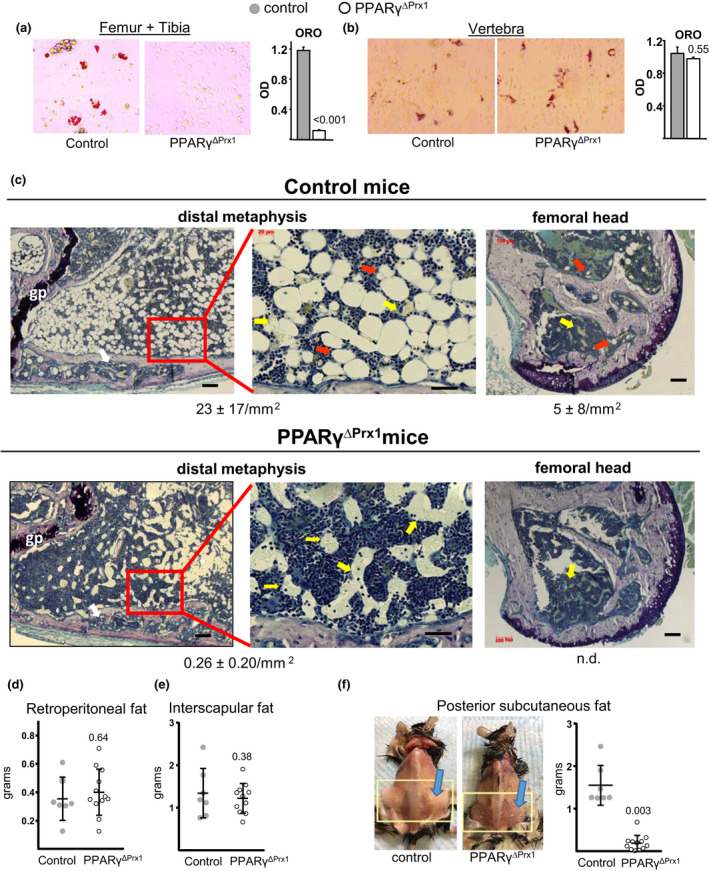
Deletion of PPARγ prevents marrow adiposity and accumulation of subcutaneous fat in aged mice. (a‐b) Oil red O staining to visualize (right panel) and quantify (left panel) adipogenesis in bone marrow stromal cell cultures from the indicated bones of 22‐month‐old female mice. Bars depict mean ± SD (c) Photomicrographs of histologic sections of the distal metaphysis of the femur near remnants of the growth plate (gp) (left and middle panels) and of the femoral head (right panels). White arrows mark sites of cortical porosity. Histomorphometric measurements of marrow adipocyte content are shown (Ad.N/mm^2^) n = 4/group, n.d. none detected). Red arrows indicate adipocytes, and yellow arrows indicate sinusoids containing red blood cells and granulocytes. Black bar = 100 µm (left and right panels) or 40 µm (middle panels). (d‐f) Weight of fat depots in 22‐month‐old female mice. (f) Appearance and weight of posterior subcutaneous fat. Data were analyzed by Students *t* test

We next investigated the impact of PPARγ deletion on the changes in bone and fat that occur between 6 and 22 months of age in female PPAR_γ_^ΔPrx1^ mice and their littermate controls. Prx1‐Cre‐mediated deletion of PPARγ in mesenchymal cells of the appendicular skeleton was maintained during aging as evidenced by the absence of a pro‐adipogenic response to rosiglitazone in cultures established from marrow of 22‐month‐old PPAR_γ_^ΔPrx1^ mice (Figure 4a). In addition, retention of rosiglitazone responsiveness in the axial skeleton due to lack of Prx1‐Cre activity was shown by equivalent levels of adipogenesis in cultures of marrow cells obtained from the vertebrae of both strains (Figure 4b). Numerous adipocytes were present in the femoral bone marrow, and a few were also observed in the femoral head of control mice (Figure 4c). In contrast, adipocytes were practically undetectable in PPAR_γ_^ΔPrx1^ mice at these sites. Despite suggestions that increased marrow adipocytes contribute to age‐dependent changes in hematopoiesis (Ambrosi et al., 2017; Zhou et al., 2017), bone marrow‐derived hematopoietic stem cells increased with age both control and PPAR_γ_^ΔPrx1^ mice (Figure S2). Likewise, the age‐dependent decrease in circulating lymphoid cells and the increase in myeloid cells were unaffected by the deletion of PPARγ (Figure S2).

Body weight and lean and fat mass of control and PPAR_γ_^ΔPrx1^ mice were indistinguishable at 6 months of age, and all three indices increased with age by a similar magnitude in both strains as determined by sequential dual‐energy X‐ray absorptiometry (DXA) measurements (Figure S3a‐c). At 22 months of age, selected fat depots were dissected and weighed. Retroperitoneal fat and interscapular fat mass were similar in the two strains (Figure 4d,e). However, there was practically no subcutaneous abdominal fat in PPAR_γ_^ΔPrx1^ mice, as determined by visual inspection and weight (Figure 4f). This is in line with previous evidence that Prx1‐Cre selectively targets subcutaneous white adipocytes but few to no brown adipocytes or visceral white adipocytes (Sanchez‐Gurmaches et al., 2015). Nevertheless, body weight and fat mass were unaffected by deletion of PPAR_γ_ (Figure S3a,b), probably because subcutaneous abdominal fat accounts for only 3% of body weight.

Deletion of PPARγ in osteoblasts and osteocytes of male mice using DMP1‐Cre has been reported to improve insulin sensitivity in young adult mice (Brun et al., 2017), but this effect was not seen in our study of aged female PPAR_γ_^ΔPrx1^ mice (that should also lack PPARγ in osteoblasts and osteocytes). Thus, fasting glucose levels were identical in both strains at 22 months of age, and there was no difference in glucose levels between aged control and PPAR_γ_^ΔPrx1^ mice at any time following a glucose tolerance test (Figure S3d).

### 2.4 Deletion of PPARγ does not influence age‐associated loss of trabecular and cortical bone

**Figure 5:**
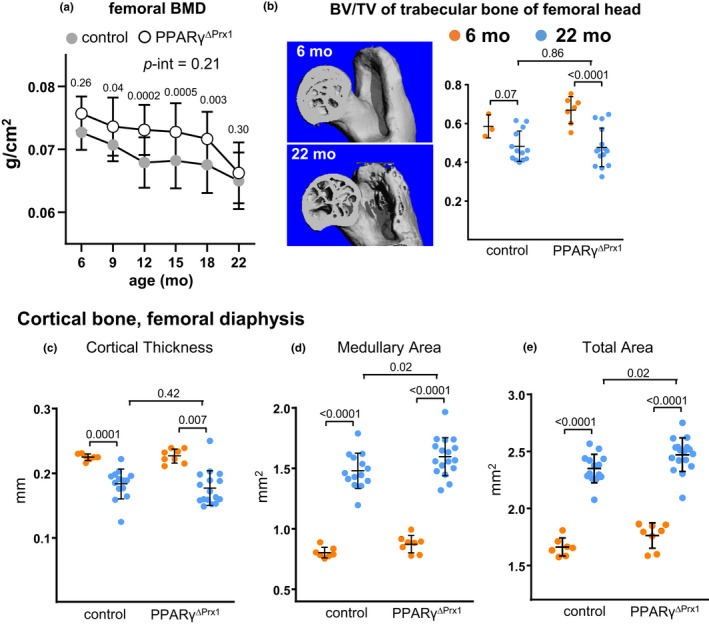
Deletion of PPARγ does not prevent age‐dependent loss of bone mass, but increases bone size. (a) Loss of femoral BMD with age in female control (n = 22) and PPAR^∆Prx1^ mice (n = 25). Data shown are mean ± SD; analyzed by RMANOVA. (b) Left panel, representative micro‐CT images of the femoral head in control mice. Right panel, BV/TV of the femoral head of 6‐ and 22‐month‐old control (n = 3, 13) and PPARγ^ΔPrx1^ mice (n = 7, 15). Some femoral heads were lost during dissection. (c‐e) Micro‐CT analysis of femoral diaphysis of 6‐ and 22‐month‐old control (n = 7, 14) and PPARγ^ΔPrx1^ mice (n = 8, 17). Data analyzed by 2‐way ANOVA

Progressive loss of femoral bone mass occurred in both control and PPAR_γ_^ΔPrx1^ female mice between 6 and 22 months of age as determined by sequential BMD determinations (Figure 5a).

Two‐way repeated measures ANOVA failed to detect an effect of PPARγ deletion (*p*‐int = 0.21). Post hoc comparisons indicated that femoral BMD was 3%–6% greater in 9‐ to 18‐month‐old PPAR_γ_^ΔPrx1^ mice than in controls (*p* = 0.04 to 0.003), but was practically identical to control mice at 22 months of age (*p* = 0.30).

Trabecular bone at the distal femur was scarce at 6 months of age in both strains. However, trabecular bone was abundant in the femoral head (Figure 5b). More important, 18% of the bone at this site was lost with age in controls versus 29% in PPAR_γ_^ΔPrx1^ mice (*p* = 0.07 and 0.001, respectively). These changes were associated with decreased Tb.N and Tb.Sp, but not Tb.Th (Table S4). Two‐way ANOVA showed little or no difference between strains in the magnitude of bone loss at this site (*p*‐int = 0.19). See Table S4 for summary of 2‐way ANOVA. At 6 months of age, deletion of mesenchymal PPARγ in females had little, if any, effect on diaphyseal cortical thickness but Tt.Ar increased by 5% (*p* = 0.13), as compared to littermate controls. The age‐dependent decline in Ct.Th at the femoral diaphysis (Figure 5c) was similar in controls and PPAR_γ_^ΔPrx1^ mice, with no statistical evidence for dependence on PPARγ (*p*‐int = 0.55). The decline in Ct.Th results from greater medullary expansion than periosteal apposition. Indeed, in both strains, the diaphyseal medullary cavity increased by 83‐84%, whereas T.Ar increased by only 40‐41%. (Figure 5d,e, Table S4). In addition, T.Ar was increased by 5% in 22‐month‐old PPAR_γ_^ΔPrx1^ mice compared to controls (*p* = 0.02) (Figure 5e). Similar findings were obtained when cortical measurements were performed at the distal metaphysis (Table S4). The increased size most likely explains the increased BMD observed up to 18 months of age (Figure 5a).

### 2.5 Deletion of PPARγ and lack of marrow adiposity exacerbate cortical porosity in aged mice

**Figure 6:**
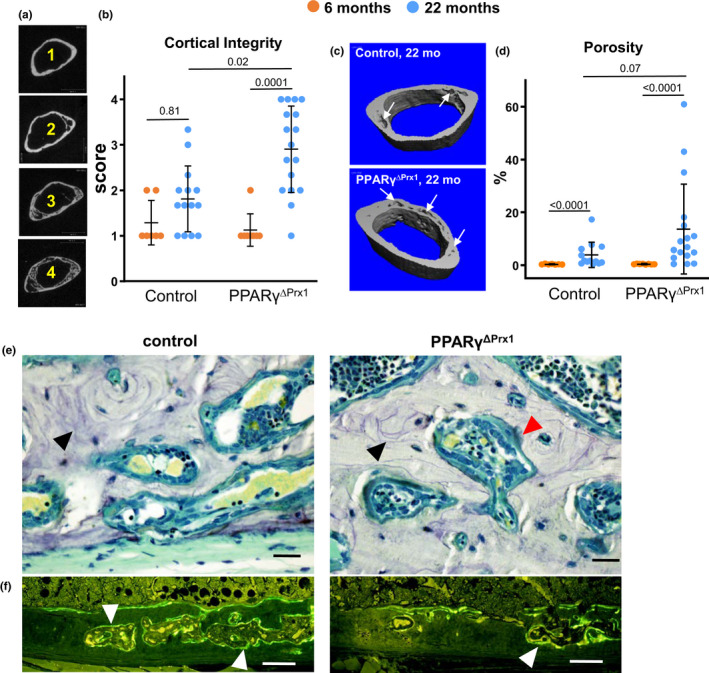
Deletion of PPARγ intensifies age‐dependent cortical porosity. (a) Cross‐sectional micro‐CT images of the femoral metaphysis from 22‐month‐old mice used to score cortical integrity: 1 = no porosity and intact endosteum; 2 = porosity with intact endosteum; 3 = porosity with moderate loss of the endosteal boundary; 4 = extensive porosity with loss of the endosteal boundary. (b) Cortical integrity score of 6‐ and 22‐month‐old control (n = 7, 13) and PPARγ^ΔPrx1^ mice (n = 8, 17). (c) Representative micro‐CT images of cortex comprising a 0.6 mm section of bone of the proximal third of the distal femoral metaphysis depicting cortical porosity (arrows), which was (d) quantified by micro‐CT. (e) Representative photomicrographs of distal femur to visualize cortical porosity; TRAPase (red arrowhead) and cement lines stained with toluidine blue (black arrowheads). Bar = 20 µm. (f) Calcein labeling (white arrowheads) in fluorescence images of unstained sections. Bar = 100 µm. Data were analyzed by 2‐way ANOVA on Ranks

We have previously documented cortical porosity in aged female mice, resulting from de novo intracortical bone remodeling (Piemontese et al., 2017). Both control and PPAR_γ_^ΔPrx1^ 22‐month‐old female mice exhibited porosity in the metaphyseal portion of the distal femoral cortex (Figure 4c, left panel). Micro‐CT imaging revealed that, in many samples, bone destruction was so extensive that the endosteal boundary was no longer evident, making it impossible to quantify porosity over the entire distal femur. Therefore, we devised an index of cortical deterioration using a single micro‐CT cross‐sectional image, located at mid‐metaphysis, and the morphologic criteria described in Figure 6a. There was little evidence of cortical bone deterioration in 6‐month‐old female mice of either strain (Figure 6b). At 22 months of age, pores were evident in 10 of the 14 control mice, but only two mice exhibited significant deterioration of the endosteal boundary. In contrast, 16 of the 17 PPAR_γ_^ΔPrx1^ mice exhibited pores at this site, and 9 of them had extensive loss of the endosteal boundary. Accordingly, the mean score increased from 2 in controls to 3 in PPAR_γ_^ΔPrx1^ mice (*p* = 0.02).

Cortical porosity was also measured by micro‐CT at the proximal third of the distal femoral metaphysis (Figure 6c), where the endosteal boundary was preserved. Porosity increased by 250% in aged PPAR_γ_^ΔPrx1^ mice as compared to controls (*p* = 0.07; Figure 6d). Consistent with this, volumetric BMD at this site declined by 10% in controls and by 14% in PPARγ^ΔPrx1^ mice (*p* = 0.001 and *p* < 0.0001, respectively; Table S4).

In an attempt to elucidate the cellular basis for the differences in porosity, we performed histomorphometric analysis of the distal femoral metaphysis from 22‐month‐old mice. As previously described (Jilka et al., 2014; Piemontese et al., 2017), the pores varied in size and contained capillaries, marrow elements, and osteoclasts. Scalloped cement lines indicative of previous episodes of intracortical bone remodeling were present, as well as fluorochrome labeling that marks sites of new bone formation (Figure 6b,c). However, there was no difference between control and PPAR_γ_^ΔPrx1^ mice in osteoclast number or mineralizing surface (reflecting active osteoblasts; Table 1). Mineral apposition rate (MAR), however, was increased by 37 ± 18% (*p* = 0.06) but this was not sufficient to affect overall bone formation rate (BFR).

**Table 1:** Deletion of PPARγ from mesenchymal cells does not influence bone remodeling in the distal femur of 22‐month‐old female mice

Parameter	Control (n = 8)	PPARγΔPrx1 (n = 7)	*p*
N.Oc/B.Pm, /mm	0.70 ± 0.44	0.74 ± 0.42	0.84
MS/BS	0.21 ± 0.05	0.21 ± 0.06	0.94
MAR, µm/day	1.21 ± 0.35	1.65 ± 0.48	0.06
BFR, µm^3^/µm^2^/day	0.26 ± 0.11	0.33 ± 0.10	0.24

Abbreviations: BFR, bone formation rate; MAR, mineral apposition rate; MS/BS, mineralizing surface per bone surface; N.Oc/B.Pm, number of osteoclast per bone perimeter.

### 2.6 The increased cortical porosity in PPARγ^ΔPrx1^ mice is not associated with intensification of osteoarthritis

**Figure 7:**
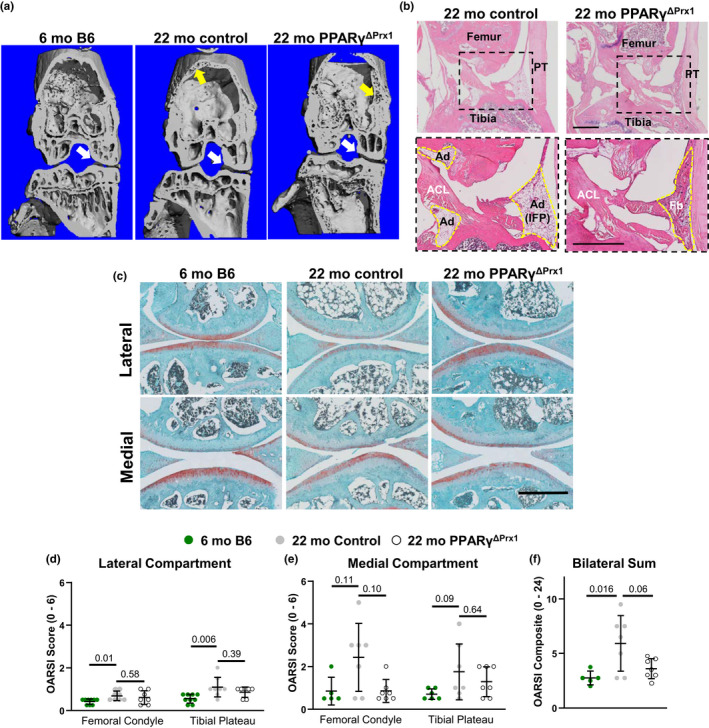
Deletion of PPARγ attenuates age‐associated osteoarthritis. (a). Representative micro‐CT reconstructions of knees in the mid‐coronal plane. White arrows indicate subarticular cortical bone, and yellow arrows mark porous cortical bone. (b) Sagittal sections of knees stained with H&E. ACL, anterior cruciate ligament; Ad, adipose tissue; Fb, fibrous tissue; IFP, infrapatellar fat pad; PT, patellar tendon. (c) Safranin O/Fast Green staining of knee joints. (d‐f) OARSI scoring in (d) lateral compartments of 6‐month‐old B6 (n = 9), 22‐month‐old control (n = 8) and PPARγ^ΔPrx1^ littermates (n = 7), (e) medial compartments (n = 5, 7, 7, respectively), and (f) the sum of all four compartments (n = 5, 7, 7, respectively). Some medial compartments could not be scored due to persistent sectioning artifacts. Bars = 500 μm. Data were analyzed by ANOVA on Ranks

In view of evidence that chondrocyte‐specific deletion of PPARγ exacerbates osteoarthritis (Vasheghani et al., 2013), we examined whether the increased porosity seen in aged PPAR_γ_^ΔPrx1^ mice was due to osteoarthritic changes in the knee. In contrast to the porosity of the metaphyseal cortex of the femur and tibia, there was little if any porosity in subarticular cortical bone of the femur and tibia of aged control or PPARγ^ΔPrx1^ mice, as visualized by micro‐CT (Figure 7a and Figure S4). Indeed, the subchondral plates of aged control mice appeared similar that of young B6 mice. Further, 3D reconstructions revealed few osteophytes in aged mice and no discernable effect of PPARγ deletion (Figure 7a and Figure S4).

Histologic examination showed that infrapatellar adipocytes, as well as adipocytes adjacent to the anterior cruciate ligament, were present in 22‐month‐old control mice but not in PPAR_γ_^ΔPrx1^ mice (Figure 7b). This finding is consistent with earlier studies in female mice (Vasheghani et al., 2013). In agreement with earlier reports (Hashimoto et al., 2017), we observed mild articular cartilage degeneration in the knees of 22‐month‐old female control mice, but not in 6‐month‐old B6 mice (Figure 7c‐f), as measured using the Osteoarthritis Research Society International (OARSI) scoring criteria (Glasson et al., 2010). Interestingly, however, scores were lower in aged PPAR_γ_^ΔPrx1^ mice compared to aged controls and were associated with reduced incidence of articular surface erosion in the medial femoral condyle. When summed over all four compartments (i.e., bilateral sum), deletion of PPARγ lowered the OARSI score by 2‐fold (*p* = 0.06) and approached that of 6‐month‐old B6 mice. Therefore, intensification of osteoarthritis cannot account for the increased cortical porosity observed in the distal femur and proximal tibia of aged female PPAR_γ_^ΔPrx1^ mice.

## 3 DISCUSSION

The results presented herein show for the first time that deletion of PPARγ in mesenchymal cells of the murine appendicular skeleton prevents the development of bone marrow adipocytes but does not affect rosiglitazone‐induced loss of cortical and trabecular bone in males, or age‐dependent loss in females, as determined in a littermate‐controlled longitudinal study. Diversion of mesenchymal progenitors from the osteoblast to the adipocyte lineage probably occurred in both situations, but the magnitude of such diversion evidently did not reduce the supply of osteoblasts enough to unbalance bone remodeling and cause bone loss. This functional genetic evidence provides a compelling argument against the hypothesis that increased marrow adipogenesis is an important culprit of TZD‐ or age‐associated bone loss, at least in the appendicular skeleton. Nevertheless, we did detect a role of mesenchymal cell PPARγ in restraining the development of age‐dependent cortical porosity. Since cortical porosity is not evident in 6‐month‐old female control or PPARγ^ΔPrx1^ mice, our findings suggest that alterations in the actions of PPARγ with advancing age normally restrain the development of cortical bone porosity in female mice. The mechanisms involved remain unknown, but appear not to involve changes in the process of intracortical remodeling. Our studies also show that increased marrow adipogenesis is not required for the increase in hematopoietic stem cells and the skewing of circulating progeny toward myelopoiesis that occurs with age (Ambrosi et al., 2017; Geiger & Zheng, 2013; Wilson et al., 2018; Zhou et al., 2017).

Intriguingly, deletion of PPARγ in mesenchymal cells increased periosteal expansion as measured by the increased periosteal perimeter and total area (reflecting increased bone size) of the femoral diaphysis in adult males and females, and this difference was maintained during aging in females. Thus, our findings strongly suggest that PPARγ restrains the modeling‐based bone formation at the periosteal surface, but not the remodeling‐based bone formation that occurs on trabecular and endosteal bone surfaces (Piemontese et al., 2017). The mechanistic basis for such differential effects of PPARγ remains unknown.

Deletion of PPARγ using the Prx1‐Cre transgene had little effect on trabecular bone volume (BV/TV) of the appendicular skeleton of young or old mice. Cao et al reported similar findings using Dermo‐Cre (Cao et al., 2020). Nevertheless, conditional deletion of PPARγ using Osx1‐Cre (Sun et al., 2013) increased femoral trabecular bone of young adult mice. Regarding effects of PPARγ deletion on cortical bone, we found an increase both total area and medullary area in the femur of aged mice, and cortical thickness was unaffected. But Cao et al., 2020 observed an increase in cortical bone thickness of the tibia due to increased total area, whereas medullary area was unaffected. Potential explanations for this discrepancy are the use of mice with a heterogeneous background (CD1 and C57BL/6J) in a cross‐sectional study design by Cao et al., 2020 as compared to our use of mice with a homogeneous C57BL/6 background in a longitudinal littermate‐controlled study design. This design minimizes the influence of factors other than age that could contribute to changes in bone mass, for example, rearing conditions, caging conditions, and other environmental influences. Another potential confounding issue is that Dermo‐Cre activity ceases between 6 and 12 months of age (Watkins et al., 2011).

Though not directly relevant to the role of PPARγ in mesenchymal lineage allocation, male mice with conditional deletion of PPARγ in mature osteoblasts and osteocytes using DMP1‐Cre exhibit increased femoral trabecular bone mass (Brun et al., 2017) and as in the present study, increased bone size. In mice‐bearing Prx1‐Cre, osteoblasts and osteocytes should also lack PPARγ, raising the question of why a similar effect was not seen in the present investigation. One possibility is that DMP1‐Cre‐mediated deletion of PPARγ in non‐skeletal cells might have indirectly affected bone mass. Indeed, recent studies have shown that a DMP1‐Cre transgene is active in muscle cells (Kalajzic et al., 2013; Lim, Burclaff, He, Mills, & Long, 2017). Deletion of PPARγ in muscle by the DMP1‐Cre transgene might also explain the improved glucose tolerance reported in 6‐month‐old mice lacking PPARγ in DMP1‐Cre targeted cells of adult male mice (Brun et al., 2017), whereas such effects were not observed in the present study using aged female PPARγ^ΔPrx1^ mice. This is consistent with decreased body weight and fat mass and increased lean mass of the former study (Brun et al., 2017), but not in the PPARγ^ΔPrx1^ mice shown herein, despite ablation of subcutaneous abdominal fat. Thus, our results argue against a role of bone marrow fat or bone mesenchymal cells of the appendicular skeleton in energy metabolism of aged female mice. Additional work is needed to determine the role of mesenchymal cell PPARγ in the bone changes seen in diabetes, obesity, and high fat diet.

The modest skeletal effects of marrow adipocyte ablation in the present study contrasts with the high bone mass seen in mouse models of lipodystrophy (Sun et al., 2013; Zou et al., 2019). Nevertheless, when peripheral, but not marrow adipocytes, are ablated, bone mass is still increased (Zou et al., 2019), arguing against a role of bone marrow adipocytes in this lipodystrophy model.

Our results clearly show that the adverse effects of rosiglitazone on the male skeleton do not depend on activation of PPARγ in cells of the mesenchymal lineage. Instead, the most likely culprits of the bone loss caused by TZDs are osteoclasts, in full agreement with the evidence that TZDs activate PPARγ in osteoclast progenitors and enhance RANKL‐induced differentiation in vitro (Wei et al., 2010; Zou et al., 2016). And, at least in some studies, TZDs increase osteoclast number in mice (Ali et al., 2005; Fukunaga et al., 2015; Lazarenko et al., 2007; Wei et al., 2010). Conversely, conditional deletion of PPARγ in osteoclast progenitors results in either no change (Zou et al., 2016) or a decrease (Wan et al., 2007) in osteoclast number.

Consistent with previous studies (Hashimoto et al., 2017), osteoarthritis was reduced by deletion of PPARγ, and if anything, it was mild in the aged control mice. In contrast to our findings, cartilage‐specific deletion of PPAR_γ_ using Col2a1‐Cre caused severe osteoarthritis in 14‐month‐old mice, associated with synovial inflammation and expression of catabolic factors (Vasheghani et al., 2013). Some of the factors that cause these changes are produced by infrapatellar adipocytes (Iwata et al., 2013), which are absent in PPAR_γ_^ΔPrx1^ mice, but are present in mice with PPARγ deficiency caused by Col2a1‐Cre deletion (Vasheghani et al., 2013). Thus, lack of adipocyte‐derived catabolic factors might explain why osteoarthritis is attenuated by deletion of PPARγ in Prx1‐Cre targeted cells. Further, our findings show that intensified cortical porosity with age in PPAR_γ_^ΔPrx1^ mice cannot be ascribed to increased osteoarthritis.

If diversion of progenitors to adipocytes at the expense of osteoblasts has little or no role in the age‐dependent bone loss, what is the significance of the widely observed phenomenon of increased marrow fat with age? We are tempted to speculate that osteoblast progenitors with DNA damage become adipocytes as part of a response to senescence signals. Several lines of evidence support a relationship between DNA damage, senescence, and the formation of marrow adipocytes. Indeed, irradiation, a strong inducer of DNA damage and cellular senescence (Chang et al., 2016), increases marrow adipocytes (Chandra et al., 2017). Conversely, genetic or pharmacologic elimination of senescent cells decreases marrow adipocytes (Farr et al., 2017). Furthermore, we have shown that osteoblast progenitors from aged mice exhibit DNA damage, markers of senescence, and a dramatic increase in PPARγ expression (Kim et al., 2017), thus potentiating their commitment to adipocytes (Almeida et al., 2009).

In view of the above, the increased cortical porosity in aged PPAR_γ_^ΔPrx1^ mice may result from the inability of damaged progenitors to become adipocytes in response to senescence. Instead, these damaged progenitors may proceed to differentiate into cells of the osteoblast lineage and are eventually incorporated into bone, further increasing the number of damaged osteocytes that are a likely source of the pro‐resorptive factors that cause porosity (Piemontese et al., 2017). Although more work is needed to investigate this notion, increased marrow adipogenesis as means of disposing of damaged mesenchymal progenitors, as proposed here, is analogous to the increased osteocyte apoptosis and the disposal of damaged osteocytes with age. We show herein and in earlier work of ours (Jilka et al., 2014) that when either of these relief mechanisms are prevented, cortical porosity is exacerbated.

## 4 EXPERIMENTAL PROCEDURES

### 4.1 Animals

Animal use protocols were approved by the IACUCs of the University of Arkansas for Medical Sciences and the Central Arkansas Veterans Healthcare System. C57BL/6J (B6) mice were obtained from the NIA‐supported colony at Harlan or from Jackson Labs (#000664, designated B6 herein), B6.129‐Pparg^tm2Rev^ (JAX # 004584, designated PPARγ^f/f^ herein), and B6.Cg‐Tg(Prrx1‐cre)1Cjt/JPrx1‐Cre mice (JAX 005584, designated Prx1‐Cre herein) were obtained from Jackson Labs (Bar Harbor, ME). Where indicated, 6‐month‐old female B6 mice were purchased from Jackson Labs as used from some experiments. Prx1‐Cre and PPARγ^f/f^ mice were interbred in house to obtain PPARγ^f/f^;Prx1‐Cre and PPARγ^f/f^ controls, which were used for experiments. Procedures for genotyping and quantification of gene copy number are provided in Supplemental Experimental Procedures. After weaning, animals were fed a diet containing 29% of calories from protein, 16% from fat, and 56% from carbohydrate (Taklad, #8640). Animals were gang caged (2–5 per cage) with same sex littermates, unless otherwise noted, and provided with paper and Nestlet (Ancare) enrichment. Euthanasia was performed CO_2_ inhalation followed by cervical dislocation.

A littermate‐controlled longitudinal study design was used. For experiments examining the effect of rosiglitazone, 150 ppm rosiglitazone (LKT Laboratories, Inc., lot catalog R5773, lot #2046344 or 2599481) was added to the diet (Teklad, # TD.150857). Animals were randomized to each diet based on their spine BMD. Single cage housing was used. Mice were pair‐fed every 2 days with 10 g of the standard chow or the rosiglitazone chow, which minimizes the hyperphagic effect of rosiglitazone (De Vos et al., 1996). Approximately 80%–90% of the food was consumed (data not shown). Females averaged 25 g at the start of the experiment and males 30 g, resulting in an average consumption of at least 24 mg/kg/day for females and 20 mg/kg/day for males.

Aging studies were done with female mice born over a 3‐month period from six cages of breeders. A subset of animals was euthanized for analysis at 6 months of age. The remaining mice were switched to Teklab global 14% protein rodent maintenance diet (Envigo, catalog 2014) containing 14% protein and 4% fat and acidified water ad libitum, to prevent excessive weight gain, and analyzed at 22 months of age. Gang caging was maintained. During this period, 4 of 32 control mice either died (n=2) or were euthanized (n=2); and 7 of 26 PPAR_γ_^ΔPrx1^ mice either died (n=3) or were euthanized (n=4). Mice were euthanized because of ocular tumors, ascites, or uncontrolled skin infections at the recommendation of veterinary personnel. As shown in Figure S5, most of these deaths occurred after 18 months of age. The survival curves were indistinguishable by the Mantel–Cox test (*p* = 0.50). The 95% CI for increased loss of PPAR_γ_^ΔPrx1^ mice was 0.2–2.2 by the Mantel–Haenszel test. Similar rates of loss of female B6 mice during aging have been reported (Arriola Apelo et al., 2016).

### 4.2 Cell culture

Osteoblast and adipocyte differentiation was analyzed as previously described (Almeida et al., 2009). For osteoblasts, freshly isolated murine bone marrow cells pooled from three mice of each genotype and cultured in 12‐well tissue culture plates at 5 × 10^6^ cells per well in α‐MEM containing 10% prescreened fetal bovine serum and 1 mM ascorbate‐2‐phosphate, for 10 days. One half of the medium was replaced every 5 days. Fetal bovine serum was then reduced to 2%, and vehicle (DMSO) or 1_ _μM rosiglitazone added to the cultures. Cultures were maintained for an additional 7 days, and 10 mM β‐glycerophosphate was added to the medium. Three days later, the mineralized matrix was stained with 40 mM alizarin red, pH 4.2. For adipogenesis, bone marrow cells were cultured in 12‐well tissue culture plates at 2.5 × 10^6^ cells per well in the medium described above for 6 days. Medium was then changed to 10% α‐MEM containing vehicle or 1 μM rosiglitazone (Sigma), and five days later, cells were fixed with 10% formalin in PBS, rinsed, and stained for 30 min with 0.15% Oil Red O (Sigma) in a 55:45 mix of isopropanol and water. Oil Red O staining was quantified after extraction of the dye with 0.5 ml isopropanol, and absorbance determined at 500 nm. For all assays, cells were plated in triplicate.

### 4.3 Body composition

Dual‐energy X‐ray absorptiometry (DXA), using a PIXImus densitometer (GE Lunar), was performed on sedated (2% isoflurane) mice, and data analyzed as we have previously described (O'Brien et al., 2005). Whole body (excluding the head) scans were used to determine lean body mass and fat mass. Scans of the entire left femur were used for measurement of BMD. For sequential determinations during aging, only animals surviving for the entire experiment were included in the final analysis.

### 4.4 Micro‐computed tomography

Bone architecture was determined on dissected femora, tibia, lumbar vertebra (L4), and knees cleaned of adherent tissue. Bones were fixed in Millonig’s phosphate buffer (Leica Microsystems) and stored in 100% ethanol. Bones were scanned with a MicroCT40 (Scanco Medical) as detailed in Supplemental Experimental Procedures Appendix S1.

### 4.5 Histology and histomorphometry

Femora were fixed in Millonig’s and embedded non‐decalcified in methyl methacrylate. For histologic characterization of the femoral bone marrow, 5‐μm‐thick longitudinal sections were cut in the medial–lateral plane positioned to include a cross section of the femoral head. Non‐decalcified sections were mounted unstained for the determination of bone formation rate. For quantification of other histologic indices, the sections were stained with 0.3% toluidine blue (Sigma‐Aldrich) in phosphate‐buffered citrate, pH 3.7. Adipocytes were identified as translucent ellipsoidal cells with a thin cytoplasmic rim separating them from nearby cells and sinusoids. These features distinguish them from capillaries and sinusoids containing marrow elements and red blood cells. Adipocyte number was determined in the marrow of distal femur starting 300 µm below the growth plate remnants and proceeding proximally to the diaphysis, defined as midway between the epiphyses. Adipocytes were also enumerated within the marrow of the femoral head. For quantification of osteoclasts, sections were stained for TRAPase as previously described (Piemontese et al., 2017) and enumerated on the endosteum and within the cortical pores of the distal femur. Fluorochrome labeling to measure bone formation was determined on intracortical and endosteal surfaces of the distal femur and presented as a combined measurement, since the endosteal surface could not be distinguished in many of the samples. Histomorphometric measurements were done using Osteometrics software. The nomenclature used was according to Dempster et al (Dempster et al., 2013). For all histomorphometric determinations, observers were blinded to the identity the sections examined.

For osteoarthritis evaluation, right knee joints, including the distal third of the femur to the proximal third of the tibia, were cleaned of surrounding muscle and fixed in 4% paraformaldehyde in PBS for 48 hr. After micro‐CT scanning, specimens were decalcified with 15% EDTA in PBS for 2 weeks and embedded in paraffin. Sagittal sections (5 µm) near the medial–lateral axis were stained with H&E to assess intra‐articular adipose deposits, while those from the medial and lateral compartments were stained with Safranin O/Fast Green/Weigert’s iron hematoxylin (Schmitz et al., 2010). Within both compartments, the articular surfaces of the femoral condyle and tibial plateau were scored for cartilage degeneration by two individuals blinded to the specimen groups, using a mouse‐specific scoring system recommended by the Osteoarthritis Research Society International (Glasson et al., 2010).

### 4.6 Statistics

Data were analyzed using SAS 904 (SAS Institute, Inc.) or Prism 8.0 (GraphPad Software, Inc.). The primary endpoints of this work are trabecular bone volume and cortical thickness. Power analysis was based on previously observed levels of variance in our laboratory in the primary micro‐CT outcomes (BV/TV and Ct.Th). It was determined that 9 animals per group are needed to observe a change of 1.6 standard deviations in these indices in 2‐way ANOVA at 80% power and α =0.05. Therefore, we aimed to use at least nine mice per group. All data are shown as mean ± SD or mean % change ± SEM as provided by Prism. The number of replicates, and statistical tests used, are provided in Figure legends and Tables. If data did not meet assumptions of normality and equivalent variance, they were either transformed or analyzed with a non‐parametric test. Exact *p* values are shown for relevant comparisons. The *p* values of post hoc comparisons after ANOVA or RMANOVA were adjusted to control the false discovery rate after the two‐stage linear step‐up procedure of Benjamini, Krieger, and Yekutieli as described in Prism 8.0. In line with the recommendations of the American Statistical Association as summarized by Amrhein et al. (2019), a threshold value of *p* was not used to define a statistically significant effect.

## CONFLICT OF INTEREST

D.Z. is co‐founder and advisor to Unity Biotechnology, which develops small‐molecule senolytic drugs for age‐related disease. The other authors have no conflicts.

## AUTHOR CONTRIBUTIONS

MA and RLJ designed the experiments. MA, HK, LH, DZ, RMP, AE, and RLJ directed experiments or carried out specialized procedures. JT performed some statistical analyses and provided advice. MA, SCM, and RLJ interpreted the data and wrote the manuscript.

## Data Availability

The data that support the findings of this study are available from the corresponding author upon reasonable request.
