# Supplementary material for: Increased marrow adipogenesis does not contribute to age‐dependent appendicular bone loss in female mice
Source: Aging Cell. 2020 Oct 13;19(11):e13247. doi: 10.1111/acel.13247 (PMC7681065; doi:10.1111/acel.13247)
Supplement: Supplementary file 1 [file ACEL-19-e13247-s001.pdf]

SUPPLEMENTAL EXPERIMENTAL PROCEDURES

Animals

Prx1-Cre and PPARγ$^{f/f}$ mice were interbred in house to obtain PPARγ$^{f/f}$;Prx1-Cre and PPARγ$^{f/f}$ controls, which were used for experiments. All mice are on a C57BL/6J (B6) background. Progeny were genotyped at weaning, and at the end of each experiment. Cre was detected using 5'-GCT AAA CAT GCT TCA TCG TCG G-3'(forward) and 5'-GAT CTC CGG TAT TGA AAC TCC AGC-3'(reverse), which yields a 650bp band (He et al., 2003). Mutated PPARγ was detected using primers specified by the Jackson Labs website: 5'-TGT AAT GGA AGG GCA AAA GG-3' (forward), and 5'-TGG CTT CCA GTG CAT AAG TT-3' (reverse), which yields a 230 bp band for the mutated gene, and a 200 bp band for the wild type gene. Full length gene (700 bp) deleted gene (300 bp) were detected using RT-PCR and the following primers: RT-PPARg-Forward: GTC-ACG-TTC-TGA-CAG-GAC-TGT-GTG-AC, and RT-PPARg-Reverse: TAT-CAC-TGG-AGA-TCT-CCG-CCA-ACA-GC, as previously described (He et al., 2003). Quantitative PCR was used to quantify gene copy number and to check for germline transmission of Cre. Custom primer designed for Exon 1 was used for this test. Sequences are: ACC-ACT-CGC-ATT-CCT-TTG-ACA (forward), TGT-CTT-CAT-AGT-GTG-GAG-CAG-AAA-TG (reverse), ACT-GTG-GTA-AAG-GGC-TTG (reporter seq). The TaqMan Copy Number Reference Assay was used to quantify intact PPARγ gene, using Tfrc as reference gene (company cat# 4458370, lot# 1704026). Osx1-Cre;TdRFP mice are the progeny of Osx1-GFP:Cre and stop-loxP-tandem dimer red fluorescent protein mice, and were obtained from our breeding colony (Kim et al., 2017).

Micro-computed tomography

Bones were scanned with a MicroCT40 (Scanco Medical) at medium resolution (12 μm isotropic voxel size) for quantitative determinations. For the latter, a Gaussian filter (sigma = 0.8, support = 1) was applied. Scanco Eval Program v.6.0 was used for measuring bone volume. Scan settings included X-ray tube potential (55 kVp), X-ray intensity (145 μA), and integration time (220 ms). Nomenclature conforms to recommendations of the American Society for Bone and Mineral Research (Dempster et al., 2013). Femora were scanned from the femoral head to the beginning of the distal growth plate. Cortical dimensions were determined at the diaphysis (18 slices, midpoint of the bone length as determined in scout view), and the metaphysis, starting 8-10 slices away from the growth plate so as to avoid the growth plate, and proceeding proximally for 151 slices, to obtain cross-sectional images drawn to exclude trabecular elements. In some experiments (as indicated), only the proximal third (50 slices) of the metaphysis was used for analysis. Cortical bone was measured at a threshold of 200 mg/cm^3. Trabecular analyses were performed on contours of the cross-sectional images drawn to exclude cortical bone and were measured at a threshold of 220 mg/cm^3. Trabecular analysis of the femoral head was made beginning at the first slice exhibiting trabecular bone, and proceeding approximately 90 slices towards the proximal growth plate near the neck. Tibiae were scanned from the proximal end to the distal tibiofibular joint; the latter 10 slices were used for measurement of cortical indices. Trabecular bone was evaluated using 100 slices from the proximal end to the tibiofibular joint. Trabecular architecture was determined using sphere filling distance–transformation indices without assumptions about the bone shape as a rod or plate.

Analysis of cortical integrity were performed using a single slice midway between the upper and lower limits of the metaphysis. Slices were scored in a blind fashion by three different observers, as follows: 1, no porosity and intact endosteum; 2, porosity with intact endosteum; 3,

porosity with loss of the endosteal boundary; 4, extensive porosity with loss of the endosteal boundary. For femoral porosity measurements, slices were analyzed from a point immediately distal to the third trochanter to a point immediately adjacent to the primary spongiosa. In some experiments, only the distal third of the metaphysis was analyzed as described above. After defining endosteal and periosteal boundaries, an additional image processing script ("peel-iter = 2") was used to eliminate false voids caused by imperfect wrap of the contours to the bone surface. Images were binarized with a threshold of 365 mg/cm^3. Cortical bone volume and void volume were determined with the "cl_image" script and used to calculate porosity. To avoid inclusion of osteocyte lacunae and canalicular space, void volumes < 31,104 μm^3 (18 voxels) were excluded in the determination of porosity.

The fourth lumbar vertebra (L5) was scanned from the rostral growth plate to the caudal growth plate to obtain 233 slices. BV/TV in the vertebra was determined using 100 slices (1.2 mm) of the anterior (ventral) vertebral body immediately inferior (caudal) to the superior (cranial) growth plate. Trabecular bone analyses were performed on contours of cross-sectional images, drawn to exclude cortical bone, as described for femoral trabecular bone. Cortical bone thickness was determined on the ventral cortical wall using contours of cross-sectional images, drawn to exclude trabecular bone, as described for femoral cortical bone.

Knees fixed in 4% paraformaldehyde were scanned with the MicroCT40 system using an energy of 70 kVp and a 300ms integration time (isotropic voxel size = 12µm), at a threshold of 200 mg/cm^3; 500 slices were collected, starting from the proximal end of patella, including the entire tibiofemoral joint, and ending in the tibial metaphysis.

Enumeration of Osx1+ osteoblast progenitors

Bone marrow cells were obtained femora and tibiae, and then depleted of CD45+ hematopoietic cells using a biotin-conjugated rat antibody specific for mouse CD45 (eBioscience, San Diego, CA, USA; 14-0451, 1:100), and three round of treatment with anti-rat IgG Dynabeads (Invitrogen, Grand Island, NY, USA) at a bead:cell ratio of approximately 4:1. Osx1-TdRFP+ cells were sorted in an Aria II cell sorter (BD Bioscience, San Jose, CA, USA) using the PE-A fluorochrome gate, as we have previously described (Kim et al., 2017).

Analysis of peripheral and bone marrow hematopoietic cells

For measurement of circulating white cells, blood samples were obtained from the orbital sinus using a micro-pipette coated with the anticoagulant K3EDTA. BM cells were flushed from mouse femurs with Hank's balanced salt solution (HBSS) after mice were euthanized by CO_2 suffocation followed by cervical dislocation. The number of various blood cells and bone marrow mononuclear cells (BM-MNCs) was counted using a pocH-100i hemocytometer (Sysmex, Kobe, Japan).

For determination of HSC and LT-HSC, bone marrow cells were flushed from femora and tibiae into HBSS containing 2% FCS, using a 21-gauge needle and syringe, and centrifuged through Histopaque 1083 (Sigma, St. Louis, MO) to isolate BM-MNCs. For the isolation of Lin- cells, BM-MNCs were incubated with biotin-conjugated rat antibodies specific for murine CD5, Mac-1, CD45R/B220, Ter-119, and Gr-1. The labeled mature lymphoid and myeloid cells were depleted twice by incubation with goat anti-rat IgG paramagnetic beads (Dynal Inc, Lake Success, NY, USA) at a bead:cell ratio of approximately 4:1. Cells binding the paramagnetic beads were removed with a magnetic field. The negatively isolated Lin- cells were washed twice with 2% FCS/HBSS and re-suspended in complete medium (RPMI1640 medium supplemented with 10%

FCS, 2 mM L-glutamine, 10 µM HEPES buffer, and 100 U/ml penicillin and streptomycin) at $1x10^6$/ml. Single HSCs (CD150+CD48-Lin-Sca1+c-Kit+ or CD34-CD150+CD48-LSK cells) were sorted by Aria II cell sorter (BD Biosciences, San Jose, CA) after Lin- cells were pre-incubated with anti-CD16/32 antibody to block the FCγ receptors and then stained with anti-Sca1-PE, c-Kit-APC-Cy7, CD150-APC and CD48-Pacific blue antibodies. Long-term HSCs (LT-HSCs, CD34-CD150+CD48-Lin-Sca1+c-Kit+ or CD34-CD150+CD48-LSK cells) were sorted by Aria II cell sorter (BD Biosciences, San Jose, CA) after Lin- cells were pre-incubated with anti-CD16/32 antibody to block the FCγ receptors and then stained with anti-Sca1-PE, c-Kit-APC-Cy7, CD34-Alex-700, CD150-APC and CD48-Pacific blue antibodies. Dead cells were excluded by gating out the cells stained positive with PI. The information for all the antibodies used is provided in Supplemental Table 5.

Glucose tolerance test

Glucose (2 g/kg body weight) was injected i.p. between 9 and 11 AM to 22-mo-old control and $PPAR^{\Delta Prx1}$ mice that had been fasted for 16 h. Blood was obtained from the tail vein measured immediately prior to glucose administration, and 15, 30, 60, 90, and 120 min afterwards. Blood glucose was determined using a glucometer (Bayer Healthcare, Contour Next).

Supplementary Table 1. Lack of effect of PPARγ deletion on femoral bone of 3-mo-old male or female mice

		**2-way ANOVA *p* values**				**female**					**male**			
	**parameter**	**sex**	**geno-type**	**int**		**Control (n=11)**	**PPARγ$^{\Delta Prx1}$ (n=9)**	**% diff (s.e.m)**	***p***		**Control (n=9)**	**PPARγ$^{\Delta Prx1}$ (n=7)**	**% diff (s.e.m)**	***p***
	Weight, g	<0.0001	0.79	0.56		19.4	19.8	2 (1)	0.55		24.9	24.8	0.4 (0.7)	0.82
Femur	BMD, g/cm^2	<0.0001	0.25	0.54		0.229	0.246	7 (2)	0.19		0.164	0.169	3 (2)	0.72
Femoral Diaphysis	Ct.Th, mm	0.25	0.52	0.26		0.207	0.209	1 (0.3)	0.71		0.214	0.209	-2 (1)	0.24
Femoral Distal Metaphysis	BV/TV	<0.0001	0.09	0.84		0.158	0.128	-19 (24)	0.15		0.275	0.252	-8 (3)	0.31
	Tb.Th, mm	0.02	0.02	0.63		0.059	0.056	-5 (5)	0.17		0.063	0.059	-6 (2)	0.07
	Tb.N, /mm	<0.0001	0.27	0.69		4.36	3.99	-8 (10)	0.25		5.54	5.42	-2 (1)	0.65
	Tb.Sp, mm	<0.0001	0.25	0.54		0.229	0.246	7 (3)	0.19		0.164	0.169	3 (2)	0.72

Data were analyzed by 2-Way ANOVA.

Supplementary Table 2. Effect of rosiglitazone in 6-mo-old male B6 mice

		**parameter**	**normal diet (n=9)**	**rosi diet (n=9)**	**% difference (s.e.m)**	***p***
		weight change, %	4.8	7.9	65 (28)	0.04
		scapular fat, g	0.29	0.57	97 (22)	0.0004
Femur	diaphysis	Ct.Th, mm	0.202	0.196	-3 (2)	0.13
	distal metaphysis	Ct.Th, mm	0.1659	0.1594	-4 (2)	0.03
	distal metaphysis	BV/TV	0.1171	0.1102	-6 (6)	0.34
	distal metaphysis	Tb.Th, mm	0.04854	0.04467	-8 (2)	0.0005
	distal metaphysis	Tb.N, /mm	4.12	4.242	3 (3)	0.35
	distal metaphysis	Tb.Sp, mm	0.2329	0.2271	-2 (3)	0.48
Tibia	tibiofibular junction	Ct.Th, mm	0.2362	0.2253	-5 (2)	0.04
	proximal metaphysis	BV/TV	0.1708	0.1609	-6 (8)	0.46
	proximal metaphysis	Tb.Th, mm	0.05422	0.05167	-5 (3)	0.09
	proximal metaphysis	Tb.N, /mm	4.666	4.767	2 (3)	0.48
	proximal metaphysis	Tb.Sp, mm	0.198	0.1968	-1 (4)	0.86
L5		Ct.Th, mm	0.06833	0.06156	-10 (2)	0.002
		BV/TV	0.2648	0.2397	-9 (3)	0.01
		Tb.Th, mm	0.05411	0.05156	-5 (3)	0.12
		Tb.N, /mm	4.841	4.888	1 (2)	0.63
		Tb.Sp, mm	0.1924	0.1921	1 (2)	0.94

Data were analyzed by t-test. rosi, rosiglitazone

Supplementary Table 3. Deletion of PPARγ has no effect on rosiglitazone-induced bone loss in 6-mo-old male mice

		2-way ANOVA *p* values				control					PPARγ$^{\Delta Prx1}$					Effect of PPARγ deletion	
	**parameter**	**diet**	**geno-type**	**int**		**ND (n=10)**	**Rosi (n=11)**	**% diff (s.e.m)**	***p***		**ND (n=14)**	**Rosi (n=15)**	**% diff (s.e.m)**	***p***		**% diff (s.e.m)**	***p***
	weight change, %	0.05	0.04	0.23		7.1	10.8	4 (2)	0.04		6.1	6.9	1 (2)	0.56		-1 (2)	0.54
	Scapular fat, g	0.001	0.39	0.13		0.69	1.10	0.4 (0.1)	0.002		0.88	1.0	0.2 (0.1)	0.13		0.2 (0.1)	0.10
**femur**																	
Diaphysis, Cortical	Ct.Th, mm	0.02	0.28	0.21		0.2038	0.2006	-2 (2)	0.44		0.2101	0.2002	-5 (2)	0.006		3 (2)	0.11
	Me.Ar, mm2	0.01	0.0003	0.33		1.069	1.116	4 (4)	0.28		1.151	1.255	9 (3)	0.0066		8 (4)	0.05
	Ec.Pm, mm	0.02	0.002	0.32		3.64	3.72	2 (2)	0.34		3.76	3.94	5 (2)	0.01		3 (2)	0.12
	Tt.Ar, mm2	0.08	0.0001	0.61		1.94	1.99	2 (3)	0.42		2.082	2.17	4 (2)	0.09		7 (3)	0.01
	Ps.Pm, mm	0.05	0.0004	0.77		4.78	4.87	2 (2)	0.27		4.96	5.08	2 (1)	0.08		4 (2)	0.02
	Vol BMD, g/cm^3	0.01	0.94	0.70		1154	1144	-1 (1)	0.15		1155	1142	-1 (1)	0.03		0.1 (0.4)	0.83
Distal metaphysis, Cortical	Ct.Th, mm	0.003	0.12	0.41		0.1759	0.1652	-6 (2)	0.013		0.1779	0.1717	-3 (2)	0.075		1 (2)	0.61
	Me.Ar, mm2	0.12	0.0003	0.78		1.245	1.32	6 (5)	0.23		1.415	1.468	4 (4)	0.31		14 (5)	0.006
	Ec.Pm, mm	0.13	0.003	0.99		4.45	4.61	4 (4)	0.32		4.76	4.92	3 (3)	0.23		7 (2)	0.04
	Tt.Ar, mm2	0.49	<0.0001	0.61		2.138	2.16	1 (3)	0.76		2.348	2.391	2 (3)	0.47		10 (3)	0.003
	Ps.Pm, mm	0.45	0.002	0.73		5.67	5.70	1 (2)	0.80		5.93	6.02	2 (2)	0.40		5 (2)	0.05
	Vol BMD, g/cm^3	0.04	0.76	0.74		1073	1058	-1 (1)	0.12		1073	1062	-1 (1)	0.19		0.1 (0.1)	0.98
	Ct.Por, %	0.20	0.55	0.83		0.47	0.57	21 (21)	0.33		0.53	0.60	13 (15)	0.39		13 (26)	0.59
Distal metaphysis, Trabecular	BV/TV	0.78	0.34	0.78		0.0874	0.08416	-4 (10)	0.72		0.0801	0.0801	-1 (10)	0.99		-8 (10)	0.4
	Tb.Th, mm	0.10	0.27	0.88		0.0462	0.0446	-3 (3)	0.23		0.0470	0.0457	-3 (2)	0.24		2 (3)	0.5
	Tb.N, /mm	0.68	0.03	0.78		3.825	3.876	1 (3)	0.65		3.687	3.697	1 (3)	0.92		-4 (3)	0.19
	Tb.Sp, mm	0.77	0.05	0.61		0.255	0.2503	-2 (3)	0.6		0.2634	0.2645	1 (3)	0.87		3 (3)	0.3
Femoral head, Trabecular	BV/TV	<0.0001	0.04	0.87		0.589	0.5448	-7 (4)	0.04		0.6082	0.5686	-7 (3)	0.02		3 (3)	0.29
	Tb.Th, mm	0.0008	0.001	0.77		0.0886	0.0787	-11 (4)	0.008		0.0945	0.0883	-7 (3)	0.024		7 (4)	0.05
	Tb.N, /mm	0.46	0.92	0.59		6.966	6.809	-2 (3)	0.41		6.888	6.862	-1 (2)	0.86		-1 (2)	0.65
	Tb.Sp, mm	0.47	0.96	0.47		0.115	0.1181	3 (4)	0.54		0.1156	0.1172	1 (4)	0.7		1 (4)	0.89

Supplementary Table 3 (continued). Deletion of PPARγ has no effect on rosiglitazone-induced bone loss in 6-mo-old males

		**2-way ANOVA *p* values**				**control**					**PPARγ$^{\Delta Prx1}$**					**Effect of PPARγ deletion**	
	**parameter**	**diet**	**geno-type**	**int**		**ND (n=10)**	**Rosi (n=11)**	**% diff (s.e.m)**	***p***		**ND (n=14)**	**Rosi (n=15)**	**% diff (s.e.m)**	***p***		**% diff (s.e.m)**	***p***
**tibia**																	
tf junction, Cortical	Ct.Th, mm	0.03	0.001	0.77		0.225	0.2196	-2 (2)	0.21		0.2356	0.2286	-3 (2)	0.06		5 (2)	0.01
	Me.Ar, mm2	0.03	0.52	0.93		0.4276	0.4565	7 (5)	0.18		0.4175	0.4487	7 (4)	0.09		2 (5)	0.61
	Ec.Pm, mm	0.08	0.34	0.63		2.39	2.44	2 (3)	0.81		2.33	2.42	2 (3)	0.36		-3 (3)	0.74
	Tt.Ar, mm2	0.31	0.19	0.84		1.112	1.137	2 (3)	0.42		1.144	1.16	1 (2)	0.43		3 (3)	0.29
	Ps.Pm, mm	0.27	0.56	0.95		3.85	3.90	1 (2)	0.42		3.88	3.92	1 (1)	0.44		1 (2)	0.65
	Vol BMD, g/cm^3	0.28	0.19	0.92		1341	1336	-0.4 (1)	0.50		1347	1342	-0.4 (0.4)	0.37		1 (1)	0.32
Proximal metaphysis, Trabecular	BV/TV	0.05	0.0003	0.70		0.119	0.1096	-8 (7)	0.27		0.0990	0.0851	-14 (8)	0.07		-17 (7)	0.02
	Tb.Th, mm	0.03	0.55	0.6		0.0519	0.0501	-3 (3)	0.27		0.0518	0.0489	-6 (3)	0.04		-1 (3)	0.96
	Tb.N, /mm	0.21	0.02	0.4		4.205	4.164	-1 (4)	0.78		4.053	3.849	-5 (3)	0.11		-4 (3)	0.28
	Tb.Sp, mm	0.21	0.01	0.36		0.2258	0.2282	1 (5)	0.82		0.2384	0.2536	6 (4)	0.10		6 (5)	0.22

		**2-way ANOVA *p* values**				**control**					**PPARγ$^{\Delta Prx1}$**					**Effect of PPARγ deletion**	
	**parameter**	**diet**	**geno-type**	**int**		**ND (n=10)**	**Rosi (n=11)**	**% diff (s.e.m)**	***p***		**ND (n=14)**	**Rosi (n=15)**	**% diff (s.e.m)**	***p***		**% diff (s.e.m)**	***p***
**vertebra, L5**																	
	BV/TV	<0.0001	0.04	0.48		0.2262	0.1927	-15 (4)	0.0005		0.2177	0.1758	-19 (4)	<0.0001		-4 (4)	0.32
	Tb.Th, mm	0.0002	0.06	0.26		0.0499	0.04812	-4 (2)	0.06		0.0494	0.0462	-6 (2)	0.0002		-1 (2)	0.58
	Tb.N, /mm	0.003	0.02	0.49		4.616	4.46	-3 (2)	0.12		4.507	4.261	-5 (2)	0.005		-2 (2)	0.25
	Tb.Sp, mm	0.0009	0.02	0.45		0.2055	0.2155	5 (3)	0.07		0.2116	0.227	7 (2)	0.002		3 (3)	0.24

Data were analyzed by 2-way ANOVA. diff, difference; ND, normal diet; rosi, rosiglitazone diet; tf, tibiofibular; int, interaction

Supplementary Table 4. Deletion of PPARγ has no effect on age-dependent femoral bone loss in females

		2-way ANOVA *p* values			control				PPARγ$^{\Delta Prx1}$				Effect of PPARγ deletion at 6 mo	
	**parameter**	**age**	**geno -type**	**int**	**6 mo**	**22 mo**	**% diff (s.e.m)**	***p***	**6 mo**	**22 mo**	**% diff (s.e.m)**	***p***	**% diff (s.e.m)**	***p***
Diaphysis, Cortical	Ct.Th, mm	<0.0001	0.73	0.55	0.225	0.1835	-18 (4)	0.0001	0.2268	0.1772	-22 (4)	0.007	1 (5)	0.87
	Me.Ar, mm2	<0.0001	0.03	0.56	0.805	1.48	84 (8)	<0.0001	0.8739	1.597	83 (6)	<0.0001	9 (8)	0.31
	Ec.Pm, mm	<0.0001	0.04	0.70	3.09	4.16	35 (3)	<0.0001	3.20	4.31	35 (3)	<0.0001	3 (3)	0.30
	Tt.Ar, mm2	<0.0001	0.008	0.82	1.663	2.351	41 (4)	<0.0001	1.765	2.471	40 (3)	0.01	6 (4)	0.13
	Ps.Pm, mm	<0.0001	0.02	0.96	4.33	5.16	19 (2)	<0.0001	4.45	5.28	19 (2)	<0.0001	3 (2)	0.14
	Vol BMD, g/cm^3	0.0007	0.95	0.73	1187	1135	-4 (2)	0.52	1192	1128	-5 (8)	0.005	1 (2)	0.87
[a]Distal metaphysis, Cortical	Ct.Th, mm	<0.0001	0.78	0.74	0.209	0.1322	-37 (4)	<0.0001	0.2058	0.1325	-36 (3)	<0.0001	-2 (4)	0.70
	Me.Ar, mm2	<0.0001	0.60	0.24	1.041	1.535	47 (7)	<0.0001	1.119	1.505	34 (6)	<0.0001	7 (7)	0.30
	Ec.Pm, mm	<0.0001	0.86	0.11	3.852	4.801	25 (4)	<0.0001	3.990	4.631	16 (3)	<0.0001	6 (4)	0.37
	Tt.Ar, mm2	<0.0001	0.04	0.95	1.987	2.257	15 (4)	0.0002	2.066	2.362	14 (3)	<0.0001	5 (4)	0.22
	Ps.Pm, mm	<0.0001	0.18	0.96	5.221	5.725	10 (3)	0.002	5.369	5.863	9 (3)	0.001	3 (3)	0.38
	Vol BMD, g/cm^3	<0.0001	0.11	0.25	1133	1024	-10 (3)	0.001	1122	963	-14 (3)	<0.0001	-1 (3)	0.76
	[b]Ct.Por, %	<0.0001	0.10	0.55	0.3	3.9	--	<0.0001	0.4	13.7	--	<0.0001	--	0.50
Distal metaphysis, Trabecular	BV/TV	<0.0001	0.33	0.26	0.0371	0.0127	-66 (13)	<0.0001	0.0306	0.013	-57 (12)	0.0002	-18 (10)	0.28
Femoral head, Trabecular	BV/TV	<0.0001	0.26	0.19	0.5843	0.4818	-18 (9)	0.07	0.6677	0.4759	-29 (6)	<0.0001	14 (10)	0.17
	Tb.Th, mm	0.90	0.13	0.44	0.0934	0.0962	3 (7)	0.69	0.1032	0.0994	-4 (5)	0.45	10 (8)	0.19
	Tb.N, /mm	<0.0001	0.81	0.26	7.122	5.064	-29 (8)	0.0007	7.59	4.76	-37 (5)	<0.0001	7 (8)	0.43
	Tb.Sp, mm	<0.0001	0.52	0.16	0.1233	0.1921	56 (20)	0.01	0.1113	0.224	101 (16)	<0.0001	-10 (22)	0.66

Data were analyzed by 2-way ANOVA. [a] Cortical architecture was measured at a 0.6 mm section of bone of the proximal third of the distal femoral metaphysis, where the endosteal boundary was preserved; [b] Porosity was analyzed by 2-way ANOVA on Ranks; mean values are shown for each genotype at 6 mo and 22 mo. Int, interaction term; The legend to Figure 5 of the main text lists animal numbers.

Supplementary Table 5. Antibodies used for flow cytometry and cell sorting

**Antibody**	**Clone**	**isotype**	**Conjugate**	**Concentration**
CD45R/B220[1]	RA3-6B2	IgG_{2a}	purified	1:200
CD3e[1]	145-2C11	IgG_{1}	purified	1:200
CD11b[1]	M1/70	IgG_{2b}	purified	1:200
Gr-1[1]	RB6-8C5	IgG_{2b}	purified	1:200
Ter-119[1]	Ter-119	IgG_{2b}	purified	1:200
CD45R/B220[1]	RA3-6B2	IgG_{2a}	biotin	1:200
CD3e[1]	145-2C11	IgG_{1}	biotin	1:200
CD11b[1]	M1/70	IgG_{2b}	biotin	1:200
Gr-1[1]	RB6-8C5	IgG_{2b}	biotin	1:200
Ter-119[1]	Ter-119	IgG_{2b}	biotin	1:200
CD16/CD32[1]	2.4G2	IgG_{2b}	Purified	1:200
CD45R/B220[1]	RA3-6B2	IgG_{2a}	APC	1:200
CD45R/B220[1]	RA3-6B2	IgG_{2a}	PE	1:200
CD11b[1]	M1/70	IgG_{2a}	PE	1:200
Gr-1[1]	RB6-8C5	IgG_{2a}	PE	1:200
Sca-1[1]	E13-161.7	IgG_{2a}	PE	1:100
c-kit[1]	2B8	IgG_{2b}	APC-Cy7	1:100
CD150[2]	9D1	IgG_{2a}	APC	1:100
CD34[2]	RAM34	IgG_{2a}	Alexa Fluor® 700	1:20
CD48[3]	HM481	IgG_{2a}	Pacific blue	1:200

[1]BD Biosciences, San Jose, CA; [2]eBioscience, San Jose, CA;
[3]Biolegend, San Diego, CA.

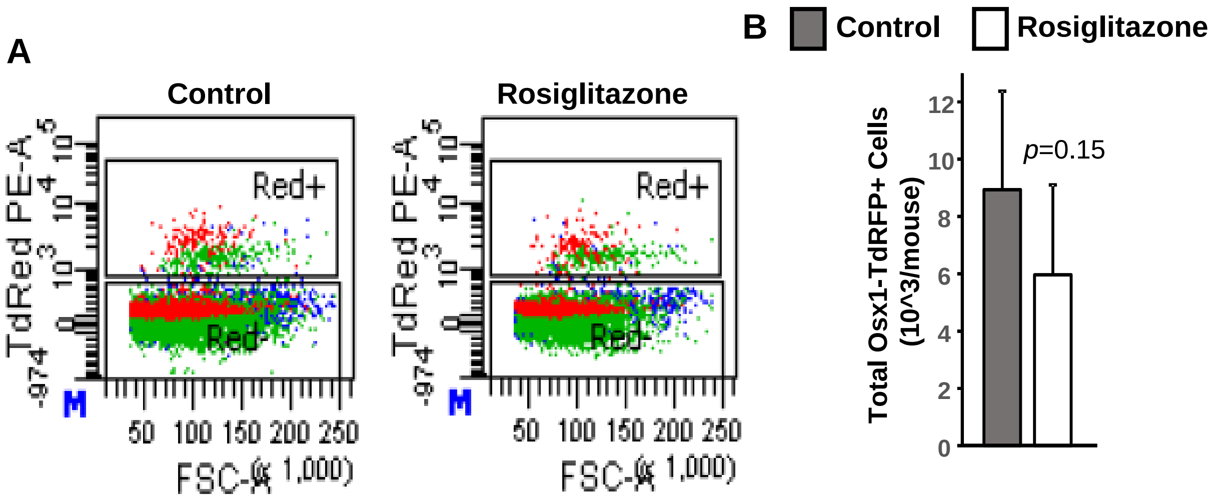

Figure S1. Rosiglitazone decreases the number of Osx1-Cre expressing cells in the bone marrow. Lineage-negative bone marrow cells were isolated from femora and tibiae of 6-month old male Osx1-Cre;TdRFP mice that had been fed control diet (n=6) or rosiglitazone diet (n=6) for 6 weeks, and then quantified by FACS. (A) Representative FACS images. (B) Number of Osx1-TdRFP+ per isolate. Data were analyzed by t-test.

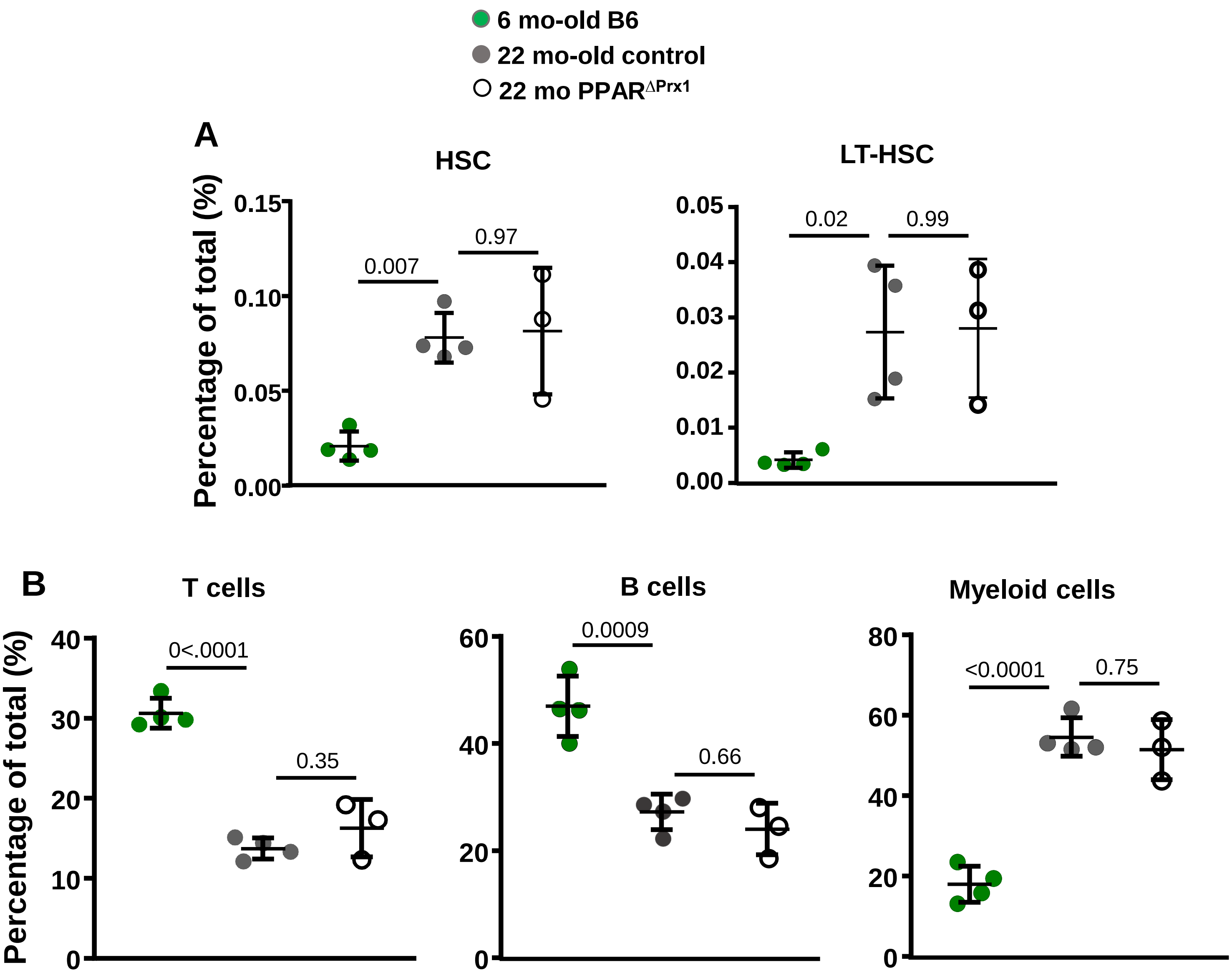

Figure S2. Deletion of PPARγ has no effect age-dependent changes in hematopoiesis. (A) The percentage of HSCs ($CD150^+CD48^-Lin^-sca1^+c\text{-}kit^+$ cells) and long term (LT)-HSCs ($CD34^-CD150^+CD48^-Lin^-sca1^+c\text{-}kit^+$ cells) in total bone marrow mononuclear cells from the indicated female mice. (B) Flow cytometry quantification of the percentages of T cells ($Thy\text{–}1.2^+$), B cells ($B220^+$), and myeloid cells ($CD11b/Gr\text{–}1^+$) in peripheral blood. Data shown are the means ± s.e.m. (*n* = 3-4 per group) analyzed by ANOVA.

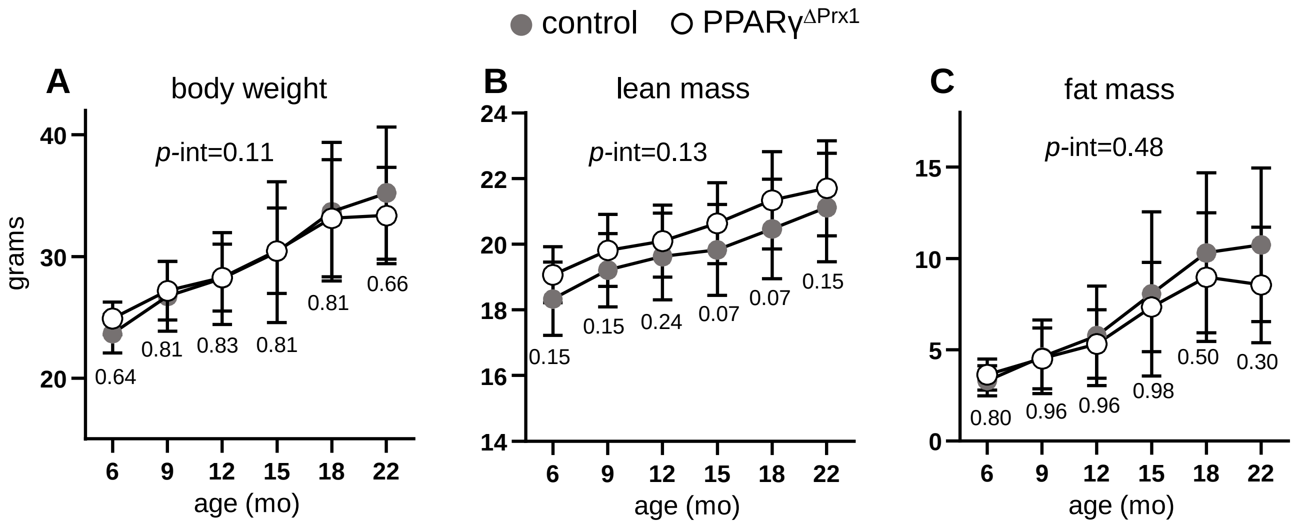

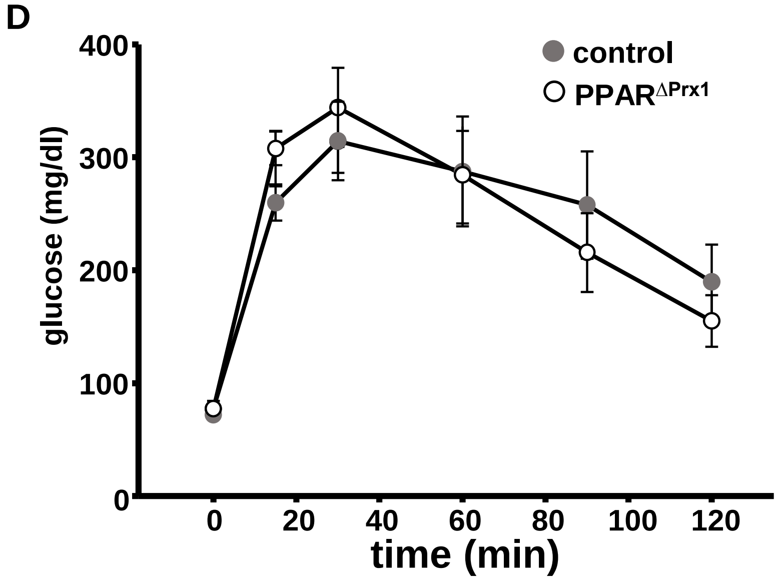

Figure S3. Deletion of PPARγ has no effect on body composition or insulin sensitivity. Longitudinal measurements of (A) body weight (B) lean body mass, and (C) fat mass of females between 6 and 22 months of age determined by DXA. (control, n=22; PPAR$^{\Delta Prx1}$, n=25). Data were analyzed by RMANOVA. Post hoc false discovery rate *p* values are shown. (D) Glucose tolerance test performed at 22 months of age in control (n=7) and PPAR$^{\Delta Prx1}$ (n=9) mice. Data shown are the mean ± sem, and were analyzed by 2-way RMANOVA. *P*>0.33 at each time point.

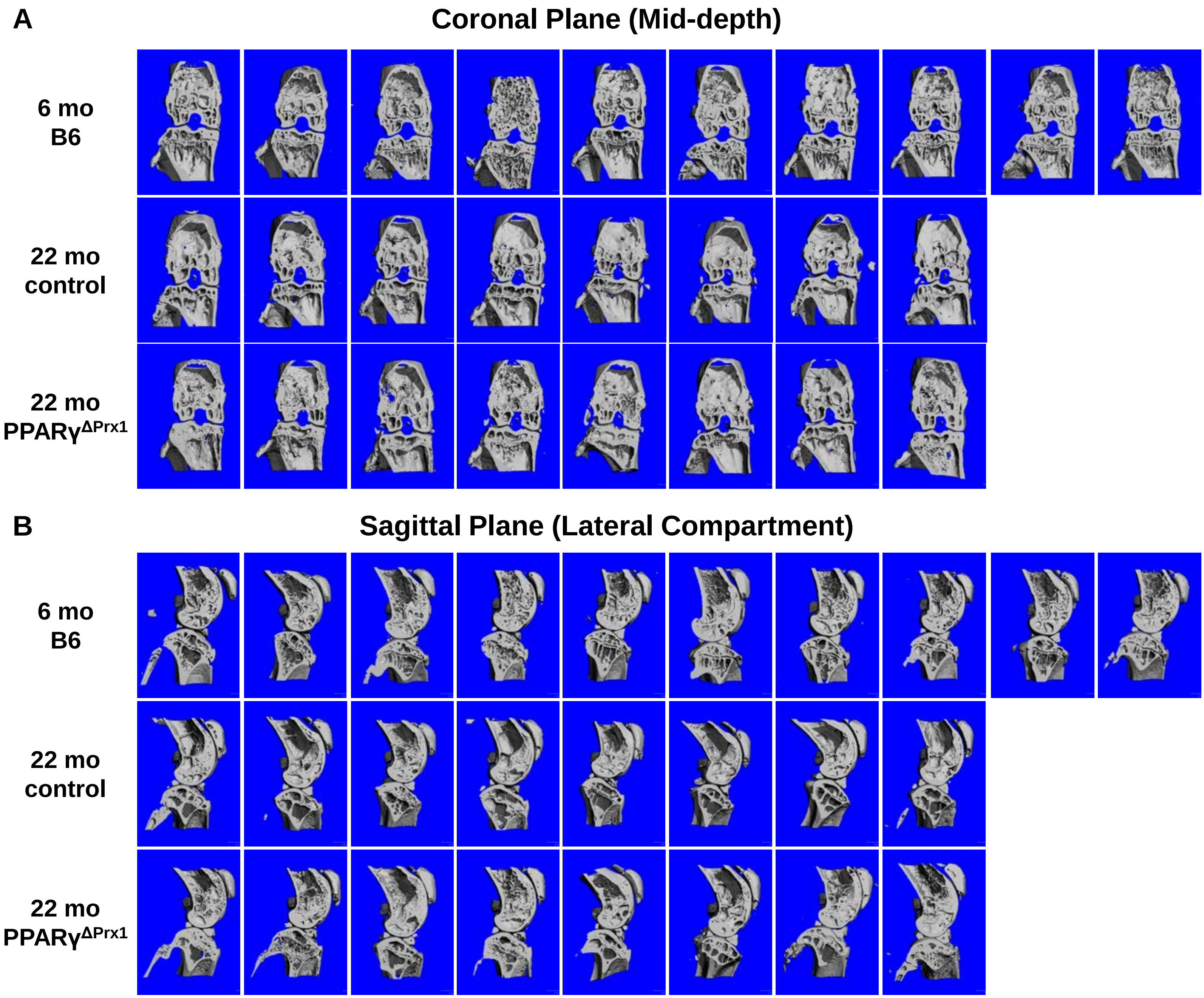

Figure S4. Deletion of PPARγ does not affect subchondral bone porosity in the femorotibial joint. 3D reconstructions of whole-knee microCT scans from the 6 mo C57Bl/6, 22 mo $PPAR\gamma^{fl/fl}$, and $PPAR\gamma^{\Delta Prx1}$ cohorts described in Figure 7. Each scan is shown from two cut planes: in a coronal plane through the anterior/posterior mid-depth (A), and in a sagittal plane through the lateral compartment of the joint (B).

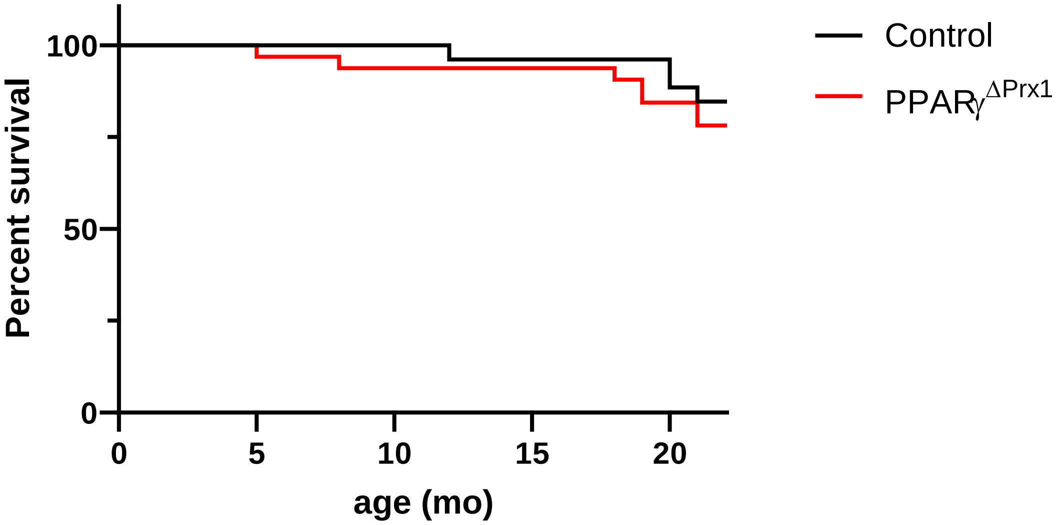

Figure S5. Survival of female control and PPARγ$^{\Delta Prx1}$ littermates. The aging study began with 32 control mice and 26 PPARγ$^{\Delta Prx1}$ mice. Data were analyzed by the Mantel-Cox test *(p*=0.50).
